# The cost of circadian desynchrony: Evidence, insights and open questions

**DOI:** 10.1002/bies.201400173

**Published:** 2015-05-22

**Authors:** Alexander C. West, David A. Bechtold

**Affiliations:** ^1^ Faculty of Life SciencesUniversity of ManchesterManchesterUK

**Keywords:** biological rhythm, circadian, clock, metabolism, obesity, SCN, shift‐work

## Abstract

Coordinated daily rhythms are evident in most aspects of our physiology, driven by internal timing systems known as circadian clocks. Our understanding of how biological clocks are built and function has grown exponentially over the past 20 years. With this has come an appreciation that disruption of the clock contributes to the pathophysiology of numerous diseases, from metabolic disease to neurological disorders to cancer. However, it remains to be determined whether it is the disruption of our rhythmic physiology per se (loss of timing itself), or altered functioning of individual clock components that drive pathology. Here, we review the importance of circadian rhythms in terms of how we (and other organisms) relate to the external environment, but also in relation to how internal physiological processes are coordinated and synchronized. These issues are of increasing importance as many aspects of modern life put us in conflict with our internal clockwork.

**Abbreviations**
FRP: free running period
SCN: suprachiasmatic nucleus

## Introduction

Life on Earth has evolved under the strong influence of daily fluctuation in the natural environment. Early organisms sequestered DNA replication to the night, so as to reduce their exposure to high energy UV light thereby reducing the mutation rate of their genetic code. Fluctuations in the environment dictate not only risks to survival, but also availability of essential resources. These include abiotic factors (e.g. light, humidity, temperature), but also biotic variables, whereby a temporal relationship with members of the same or different species (determining sociality, mate selection, predator avoidance, and prey availability) are paramount to survival and reproductive success 1, 2. Thus, as ecosystems became more complex, organisms exploited not only physical niches within their surroundings but also temporal ones 3. This has ultimately led to a world in which almost all forms of life compartmentalize biochemical, physiological, and/or behavioral processes to specific times of the day. Orchestration of these rhythms falls in large part to internal timing systems known collectively as the circadian clock. As any good time‐keeping mechanism, circadian timing systems are robust, self‐sustaining, and predictive (i.e. relevant) for the external environment. Therefore, when placed under constant conditions biological clocks of most organisms continue to track near 24 hours time reflective of the external diurnal world.

The molecular framework of circadian timing is similar in all organisms, however, there is no apparent genetic conservation between the specific components of prokaryotic and eukaryotic circadian clocks, suggesting that selective pressure drove the evolution and propagation of biological timing on multiple occasions 4. This implies a clear selective advantage associated with internal timing, which transcends the complexity of the organism and its ecological niche. Interestingly, recent work has revealed a rhythmic cycle in the oxidation state of peroxiredoxin proteins (a family essential to the inactivation of damaging reactive oxygen species) that does not depend on de novo transcription or translation, and which is conserved between archaea, bacteria, and eukaryotes 5, 6. This implies a lineage of some 2.5 billion years, and suggests that circadian rhythmicity has conferred an evolutionary advantage since bacteria first became able to photo‐dissociate water (thereby producing the selective pressure of atmospheric oxygen) 7. These findings also suggest that cycles in cellular redox state may have formed the basis upon which all subsequent genetic clocks were built.

In order to accurately predict and therefore anticipate fluctuations in the environment, internal clocks must stay linked (entrained) to the world around it. To stabilize internal and external coherence, biological clocks remain responsive to the environment through the influence of entraining factors (called zeitgebers, from the German “time‐giver”), which can influence or reset the clockwork much like adjusting a wristwatch. The daily cycle of light and dark is the most conspicuous of environmental fluctuations, and as such is the dominant zeitgeber for most organisms. However, many other signals can also have a strong influence on the clockwork, such as food availability and temperature. The type and relative strength of different zeitgebers will be organism‐, environment‐, and (in higher organisms) cell‐ or tissue‐specific. Nonetheless, when optimally aligned with the environment, the circadian system exists in a state of resonance whereby the internal clock reflects and is constantly reinforced by the world around it.

As human societies have progressed and exerted ever‐increasing control over our lifestyle and surrounding environment, this natural framework of perpetual and predictable rhythmicity has been undermined. Hyper‐connectivity, globalization, and international business markets now preclude our evolved expectation to exist solely within our local time zone. We typically view our ability to travel, work, and communicate without heed of time of day or distance as a great advance. However, it is becoming hard to ignore the evidence that erosion of our circadian rhythms is associated with a collection of metabolic problems including obesity, diabetes, and metabolic syndrome, as well as other disorders involving immune dysfunction, neurobehavioral abnormalities, and cancer 8, 9, 10. In this review, we briefly discuss the role of the circadian system in coordinating behavioral and physiological processes both internally, and in relation to the external environment. We then consider how circadian processes may be undermined in the context of modern society, and whether loss of circadian timing is detrimental to our well‐being.

## Coordination of our physiology by the circadian clock: Internal and external synchrony

**Figure 1:**
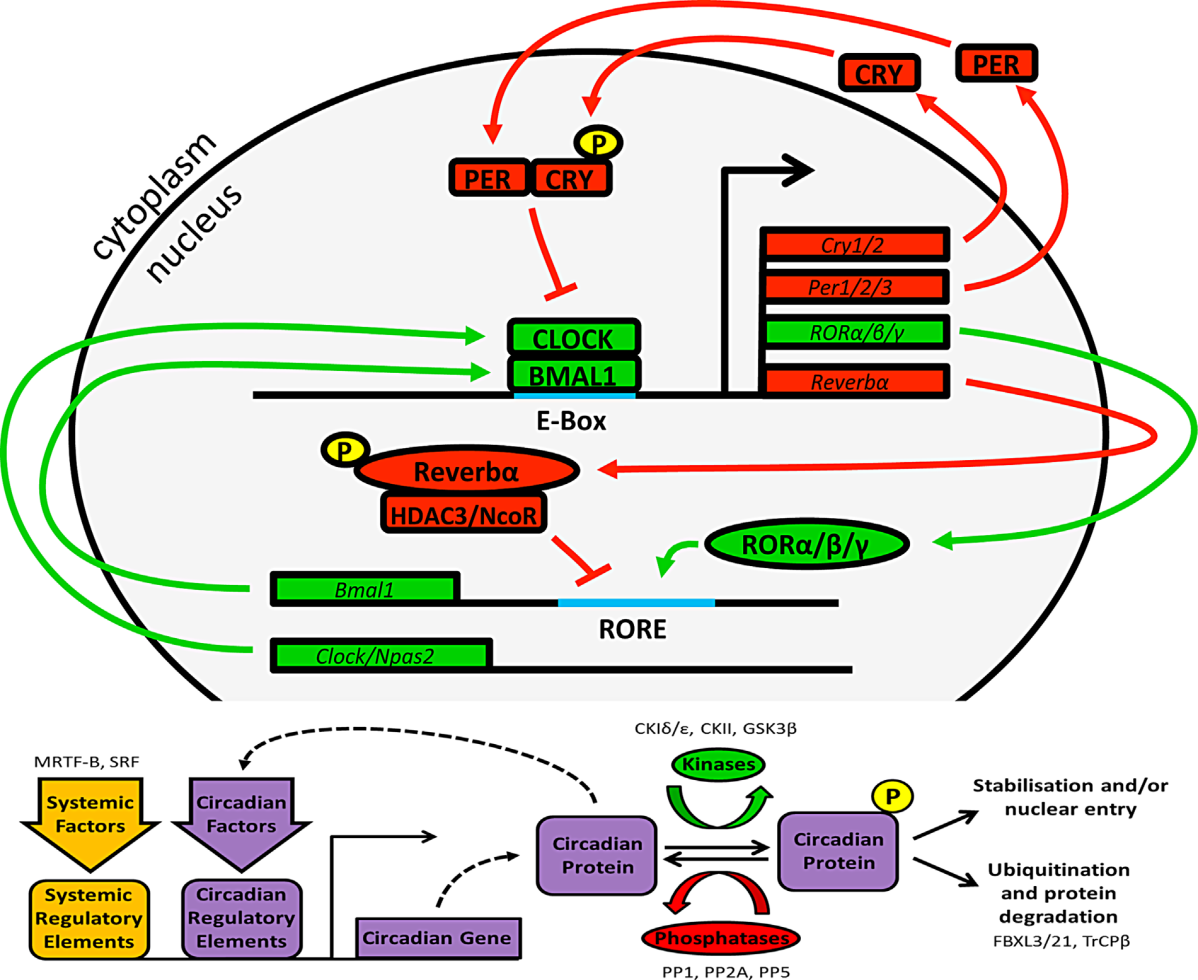
The molecular clockwork in mammals. In mammals, the molecular basis of circadian timing involves a transcriptional/translational/post‐translational feedback loop centred on the transcriptional activators CLOCK (or NPAS2) and BMAL1 and repressors PERIOD (PER1,2,3) and CRYPTOCHROME (CRY1,2). CLOCK and BMAL1 heterodimers bind to E‐box recognition sites to drive transcription of Period (Per1/2/3) and Cryptochrome (Cry1/2) 11, 12, 13, 14, 15, 16. PER and CRY proteins subsequently interact to form the core of a repressive complex, which inhibits BMAL1:CLOCK activity and expedites their clearance. Subsequent reduction of transcriptional activity at the Per and Cry loci, combined with active targeting of PER and CRY proteins for ubiquitination and degeneration (by TrCPβ and FXBL3/21, respectively) attenuates this repressive arm of the clock 116. CLOCK and BMAL1 concentrations rise once again to perpetuate the cycle. This core loop is influenced by auxiliary feedback loops, such as that involving the nuclear hormone receptors REV‐ERBα and RORα/β/γ 117, 118. Circadian proteins are also highly regulated by kinases and phosphatases which affect their stability, turnover, and sub‐cellular localization. Critically of course, components of the clock do not only drive their own expression cycles, but also impose rhythmic expression profiles onto a vast array of target genes through transcriptional enhancer element activity (e.g. via E‐Box and RORE elements), rhythmic chromatin modification (e.g. via CLOCK driven acetylation), and interaction with other non‐clock transcription factors (e.g. PER inhibition of PPARγ). In this way, transcriptional and biochemical processes within the cell are temporally coordinated across the cycle. Finally, many circadian factors are also responsive to systemic factors, which drive local cellular signaling pathways to achieve global synchronization of circadian phase.

The molecular basis of circadian timing in mammals is provided by transcriptional/translational feedback loops centered on the transcriptional activators CLOCK and BMAL1, and repressors PERIOD (PER1,2,3) and CRYPTOCHROME (CRY1,2) (see Fig. 1). This core molecular clock cycles with a near 24‐hourly periodicity, and drives temporally orchestrated waves of gene transcription 11, 12, 13, 14, 15, 16. There are functional circadian molecular clocks found within almost every cell of the body; however to establish coherent behavioral and physiological rhythms, circadian timing systems exhibit a structured anatomical organization through a specific hierarchy of pacemakers and slave oscillators 17. Here, distinct cell populations exhibit particularly robust expression of the molecular clockwork, and impose this temporal information onto local, systemic and/or behavioral processes.

In mammals, the dominant clock is generated within the ∼20,000 hypothalamic neurons of the suprachiasmatic nucleus (SCN) 18. The SCN is exquisitely sensitive to environmental light through direct connection with the retina. Classic photoreceptors and photoreceptive retinal ganglion cells project heavily to the ventral lateral SCN via the retino‐hypothalamic tract 19, 20, 21, 22. Immediate early genes, including Per1, are strongly induced in response to light, and consequently influence the phase of the molecular clock rhythm within the SCN 23, 24. Depending on the timing of light exposure, the phase of the SCN clock can be advanced or delayed. In this way, the master SCN clock, and in turn behavioral rhythms of the animal, remains closely synchronized to the prevailing environmental light‐dark cycle. Individual SCN neurons exhibit high amplitude expression of the molecular clock components, and maintain self‐sustained rhythmicity in terms of both gene expression and neuronal activity 25. SCN neurons also exhibit a high degree of inter‐cellular coupling, thereby forming a synchronous multi‐oscillator network capable of producing robust and high‐amplitude rhythms. The importance of the SCN in dictating rhythmicity is demonstrated by the fact that destruction of the SCN renders an animal behaviorally arrhythmic 26, 27.

Projection pathways from the SCN are well described, and include many hypothalamic nuclei involved in the regulation of sleep, arousal, thermogenesis, feeding behavior, as well as autonomic, and neuroendocrine output pathways 28, 29. Through such connections, the SCN imposes temporal gating to homeostatic responses of the hypothalamus, as well as drives the rhythmic release of hormonal signals such as secretion of melatonin from the pineal gland and adrenal production of glucocorticoids. In addition to the SCN, semi‐autonomous circadian clocks run in most cells and tissues of the body. Impressively, SCN transplant to a previously lesioned animal is capable of restoring behavioral rhythmicity, as well as re‐establishing rhythmic expression of gene expression in some peripheral tissues (liver, kidney), but not others (heart, spleen) 27. Murine parabiosis studies have provided similar evidence, whereby SCN‐lesioned animals maintain rhythmic phase of the molecular clock in liver and kidney when conjoined with an intact partner 30. These studies highlight the importance of both systemic factors and direct neuronal contact in dissemination of SCN‐based timing information across the body.

## Internal coordination and synchrony

In addition to providing continuity between the external and internal environments, the circadian system both synchronizes and coordinates the relative phasing of a multitude of diverse internal physiological processes and tissue systems 31. Such internal coordination optimizes responses to fluctuation in our physiological state (such as daily cycles in feeding and fasting), and strengthens homeostatic control mechanisms. For example, regulatory pathways involved in lipid metabolism and glucose homeostasis are under tight circadian control 32, 33, 34, 35, and coordination of these processes in metabolic tissues such as the liver, muscle, and adipose tissue ensure that energy supply remains relatively constant across the day and night. Despite their subservience to the SCN, local tissue clocks are critical to rhythmic gene expression in the tissue, and in many cases, overall tissue function. For example, genetic ablation of the biological clock selectively within the liver attenuates the expression of glucose regulatory genes, and compromises systemic glucose homeostasis 33. The influence of the local clockwork is not surprising given that gene array studies in mice demonstrate that in most tissues, at least 10–15% of all cellular transcripts oscillate in a circadian manner 34, 35, and >43% of all protein encoding RNAs cycle in at least one tissue 36.

Peripheral clocks do not have the same level of connectivity as the SCN oscillator, and are synchronized by the SCN through a combination of neural and humoural signals (as discussed above) 27, 37, 38. Thus overall timekeeping occurs through a network of clocks kept in synchrony by the SCN 17, 39. An important interaction exists between systemic entraining signals and components of the local clockwork in shaping the transcriptional landscape within a given tissue. For example, Kornmann et al. found that while disabling the liver clock removed the rhythmic expression profiles of the vast majority of oscillating genes, a subset retained a robust circadian rhythm in expression 40. Compellingly, one of those rhythmic factors unaffected by the loss of the hepatic clock was Per2; perhaps not surprising, given that the Per2 promoter contains many non‐circadian regulatory elements responsive to systemic signalling factors such as glucocorticoid and serum response factor 41, 42. However, Per2 is a core element of the clock and robust rhythmic Per2 transcription and translation persists in cultured liver tissue. Therefore, Per2 is influentially positioned at the interface between the systemic signalling and the local tissue clock 40.

SCN‐driven rhythms in major physiological processes such as arousal, body temperature, and feeding behavior reinforce and consolidate clock synchrony across the multitude of body clocks. Both temperature and food intake are potent entraining stimuli for peripheral circadian oscillators 43, 44, 45; so much so that peripheral clocks can become decoupled from the light‐driven SCN, when food intake is desynchronized from normal daily patterns of activity. This has been extensively modeled in rodents using restricted feeding schedules (RFS), whereby access to food is limited to a time‐window that is out of phase with the usual activity period (e.g. during the day in nocturnal rodents). Under RFS, numerous physiological and metabolic functions become entrained to the availability of food, e.g. locomotor activity, insulin, and corticosterone release. RFS has relatively little influence over the SCN which remains locked to the light cycle; yet clocks within many peripheral tissues including liver, kidney, heart, and pancreas transpose readily to reflect the timing of food intake 45, 46. In addition to the direct action of consumed nutritive factors, peripheral clocks are responsive to a range of energy‐related hormones (e.g. glucocorticoids, insulin, ghrelin, leptin) and signalling pathways (e.g. SIRT1, PPAR, AMPK) 47. The strong influence of metabolic pathways on the molecular clockwork suggests that abnormal energy supply (such as feeding schedules that are out of synchrony with normal patterns of behaiour) will undermine circadian rhythmicity.

## From internal coherence to circadian disruption: The advent of modern life

**Figure 2:**
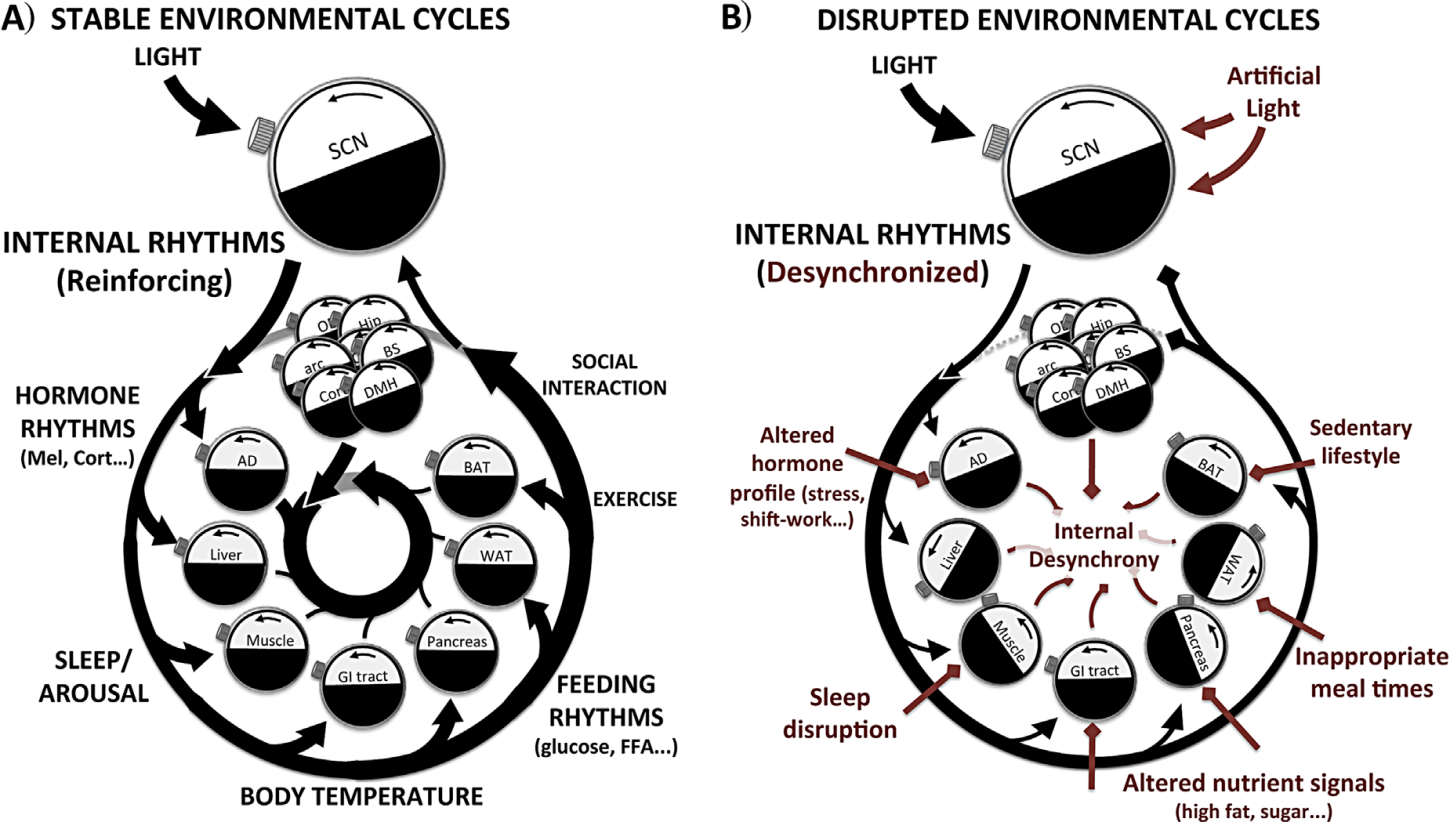
Consolidation of internal and external entraining signals to reinforce internal synchrony. **A:** The master SCN clock is powerfully synchronized by light, the most conspicuous and predictable fluctuating environmental factor. The SCN imparts this information to the rest of the body, via neural contacts to other brain clocks, and certain peripheral tissues. Importantly, alignment to the SCN rhythm is reinforced across the body through complimentary and consolidating rhythms in hormone release (most notably, melatonin from the pineal, and glucocorticoids from the adrenals), body temperature, and feeding behavior. Further consolidation will come from subordinate brain or peripheral tissue clocks, whose activity is both entrained by the SCN, but also propagates the rhythm by influencing rhythmic physiology (e.g. arousal pathways in the brain) or the rhythmic production of secreted factors which themselves can feedback and influence the phase or amplitude of the clockwork. Therefore when properly aligned, the circadian system exists in a state of resonance whereby internal clocks are constantly reinforced by our environment and behavior. **B:** Many aspects of modern life undermine the temporal stability and consolidation of internal and external zeitgebers. Should zeitgebers arrive at a phase interval that is highly variable or that does not match that of the clock, biological clock‐driven rhythms would be continually phase reset and be unable to achieve the optimal alignment with the environment. This would undermine the predictive ability of the clock, whereby biological processes become only passively responsive to environmental change, or worse unable to adequately respond due to misalignment.

Importantly, alignment of clocks across the body to the SCN rhythm is reinforced through complimentary and consolidating rhythms in hormone release (most notably, glucocorticoids from the adrenal glands), body temperature, and feeding behavior. Further consolidation will come from subordinate brain or peripheral tissue clocks, whose activity is both entrained by the SCN, but also propagates the rhythm by influencing rhythmic physiology (e.g. arousal pathways in the brain) or the rhythmic production of secreted factors which themselves can feedback and influence the phase of the clockwork. For example, insulin and leptin release from the pancreas and adipose tissue (respectively) is influenced by the local tissue clocks, as well as fluctuations in feeding/fasting cycles, yet have also been shown to themselves act upon the molecular clockwork outside their tissue of origin. Finally, circadian organization of behavior also serves as a potential reinforcing stimulus. For example, exercise, social interaction, and feeding are almost exclusively sequestered to “active” phases of the circadian cycle. All of these activities are well established entraining signals for peripheral clocks. Therefore, internal coherence of our circadian clocks, and circadian rhythms as a whole, are normally reinforced by temporal coordination of numerous internal and external rhythmic signals (as depicted in Fig. 2), and when properly aligned, the circadian system exists in a state of resonance whereby internal clocks are constantly reinforced by our environment and behavior.

Should zeitgebers arrive at a phase interval that does not match that of the clock, rhythms would be continually phase reset and be unable to achieve the optimal alignment with the environment. This would undermine the predictive ability of the clock, whereby biological processes become only passively responsive to environmental change, or worse unable to adequately respond due to misalignment. Unfortunately, it is now relatively common for the temporal coherence of our environment to be disturbed (e.g. variable meal‐times, nocturnal light exposure from artificial ambient lighting and electronic devices), and to experience sudden shifts in the phase relationship between our internal clocks and the environment (e.g. during shift‐work, jet‐lag).

The impact of rotating shift work on our circadian system is more overt, and has been associated with an abundance of pathologies, including type‐2 diabetes 8. Similarly, in animal studies, acute or repeated shifts of the light/dark (LD) cycle have been associated with enhanced tumor progression, altered immune response, cardiovascular pathology, as well as increased mortality in aged mice 48. We also know from animal studies, that different tissue clocks adjust to shifts in the LD cycle at different rate, with the SCN exhibiting the fastest rate of re‐entrainment lagged by peripheral organ clocks 49. Differences in the shift rate of different tissue clocks means that any abrupt shift will be accompanied by a period of internal desynchrony. Unfortunately, it is now commonplace to endure regular bouts of jet lag and shifting work schedules, with 15–20% of Europeans and Americans work irregular or shifting schedules 50. As impactful perhaps are the incongruous social and professional schedules that many of us now lead, which cause regular periods of sleep deprivation, exposure to light at inappropriate times, and “social jet lag” whereby we operate outside the phase of our endogenous circadian rhythm.

Despite the association of circadian disruption with disease incidence often reported in observational studies in human populations, and a flood of reviews on the subject within the scientific literature, it remains unclear whether disconnection from our environment or even loss of our internal rhythmicity has a profound impact on our health and well‐being. In the next section, we discuss whether there is any inherent value of circadian rhythmicity.

## Is there an inherent benefit to having a biological clock?

The adaptive benefits of biological timing and circadian organization are easy to conceptualize, especially in the context of a highly rhythmic external environment. Studies involving mutation or deletion of components of the clock in laboratory animals almost unanimously support a view that normal clock function is important to well‐being 51, 52. For example, CLOCK mutant mice develop a broad syndrome of metabolic dysregulation and neurological disturbance 52, 53; Bmal1 knockout mice are infertile, have diminished organ and body size and display an advance in age‐related characteristics and increased oxidative stress in the kidney, heart, and spleen 54, 55; Per2 deficient mice show increased bone mass, and are prone to cancer 56, 57, 58; and Reverbα knockout mice are obese, exhibit altered lipid metabolism, and aberrant thermogenesis 59, 60, 61. However, there is no single trait common to all clock gene mutations, and some pathology can be ameliorated through non‐rhythmic rescue of the affected clock gene (i.e. it is the presence of the factor, and not its cyclical expression that is important) 62. Therefore, it is uncertain whether pathological phenotypes result from circadian disruption per se, rather than the loss of other pleiotropic actions of the gene. This raises the question of whether there is an inherent value to circadian rhythmicity, which goes beyond the individual genetic components of the clockwork. This has been a longstanding and fundamental question in circadian biology, yet providing concrete evidence has proved complicated, especially in higher organisms such as mammals.

If the internal circadian clock does not possess an intrinsic value, it follows that organisms inhabiting aperiodic environments would exhibit a regression of their circadian phenotype due to the lack of selective pressure. Several studies have assessed the pervasiveness of the circadian rhythm in *Drosophila melanogaster* by maintaining them in constant conditions of light, heat, food, and humidity for hundreds of generations 63, 64, 65, 66. Each of these trials saw the flies preserve their circadian behavior, indicative of an intrinsic adaptive value of circadian rhythms. However, this has not always been the case when studying animals in a natural environment. For example, evidence suggestive of circadian regression in non‐rhythmic environments has been collected from studies of some cave dwelling and artic species. When monitored under constant conditions, such organisms often show weak behavioral rhythms that vary greatly in period (ranging from 10 to 57 hours per cycle) 2. A recent study of the cave fish species *Astyanax mexicanus* demonstrated a preservation of its ability to entrain to a 12:12 hours LD cycle in terms of behavior and molecular rhythms in Per1 expression 67. However, these animals become immediately arrhythmic when transferred to constant dark (DD) and fail to display rhythmic or light dependent transcriptional responses in other clock factors including Per2. It appears that the fish have evolved a tonically active light‐dependant signalling pathway, which includes Per2, linked to an up‐regulation of DNA repair machinery. Therefore, despite retaining functional clock machinery, evolution of *Astyanax* in DD has tempered circadian rhythmicity in favor of other light‐responsive pathways. Arctic species also provide a unique opportunity for studying circadian adaption to prolonged periods of constant light or darkness. For example, reindeer and ptarmigan do not show overt circadian rhythms in activity during periods of constant condition 68, 69, 70, 71. In vitro analyses of reindeer clocks suggest that molecular rhythms are profoundly attenuated, supporting a view that circadian mechanisms have regressed in this species due to lack of evolutionary pressure 72. Although it is possible that weak circadian clocks are typical of ungulate species in general 73, these studies suggest that in arrhythmic environments any inherent value of circadian rhythms may be outweighed by the advantages of a relatively tonic biology. Similarly a study using regionally distinct species of teleost fish revealed an inverse correlation between the strength of circadian rhythmicity and latitude 74. These data suggest that fish inhabiting areas with seasonal changes in their light cycles have weaker clocks and are more responsive to ambient illumination to inform their behavior. However, many other species do not appear to forgo rhythmicity in seemingly arrhythmic environments. This may of course be due to the perseverance of subtle or as of yet unrecognized zeitgebers, or that insufficient time has passed for circadian mechanisms to have eroded from the genome.

Another approach to assess the intrinsic adaptive value of circadian biological rhythms is to reduce the clock's ability to relate to (i.e. achieve resonance with) environmental cycles. By challenging the clock with non‐resonant (i.e. non‐24 hours) environments, these laboratory experiments allow an examination of the adaptability of the clock, but also the impact of inappropriate circadian timing of the biology of the organism. Indeed, studies in non‐mammalian species have shown that resonance with the environment confers a clear competitive advantage. Early studies by Ashoff and Pittendrigh demonstrated that the longevity of *Drosophila melanogaster* (fruit fly) and *Phormia terraenovae* (blow fly) was significantly reduced when the insects were housed under non‐24 hours LD conditions (e.g. 22 or 27 hours cycle length with symmetrical light/dark periods) or constant light in comparison to their natural 24 hours regime 75, 76. Similarly, studies involving *Sarcophaga argorostoma* (flesh fly) and Componotus ants demonstrated that development of the insects is delayed when maintained under non‐24 hours symmetrical LD cycles, implicating a photo‐entrainable clock as important for dipteran and hymenopteran developmental processes 77, 78. These findings have been mirrored in plants species, which show greatest growth under 24 hours lighting cycles 79.

**Figure 3:**
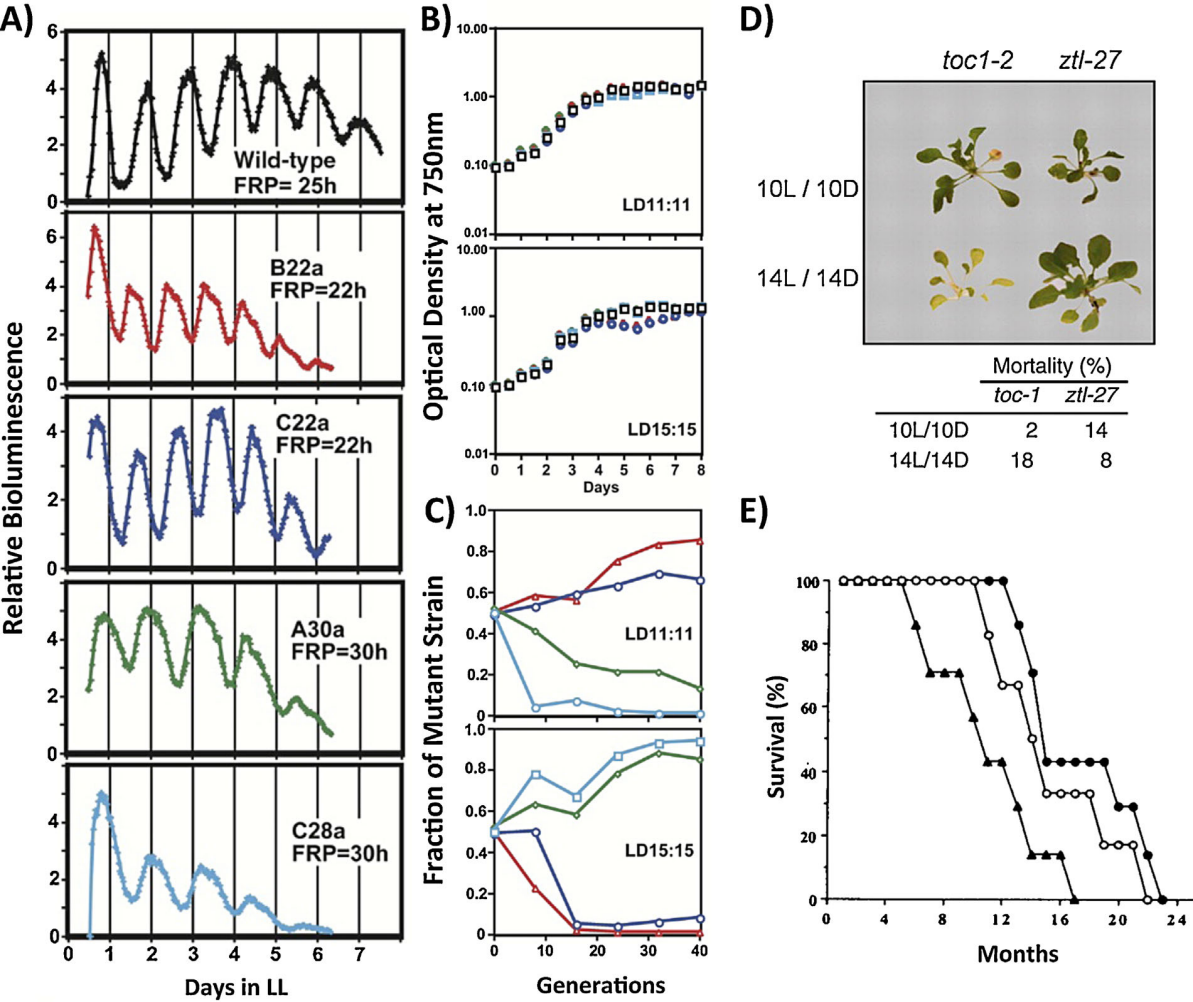
Selective advantage is conferred by resonance between internal clocks and environmental cycles. Evidence for the inherent benefit of circadian resonance. **A–C:** Competition in mixed cultures of S. elongatus. **A:** Free running periods (FRP) of wild type and circadian mutant strains. **B:** Growth of each strain in individual cultures under LD 11:11 and LD 15:15 **C:** Competition kinetics in mixed cultures of wild type and mutant strains under LD 11:11 and LD 15:15. Data plotted as proportion of mutant strain in mixed versus the estimated number of generations. Strains whose FRP match the LD cycle outcompete the wild type strain, conversely strains whose FRP is dissonant with the LD cycle are at a competitive disadvantage 81. **D:** Two representative *A. thaliana* circadian mutant strains (toc‐1, FRP 20.7 h; ztl‐27, FRP 27.1–32.5 h) from a competition experiment. Lower mortality is observed in the line whose endogenous clock matches the light environment 82. **E:** Survival plots from wild type (FRP 24 h, filled circles), heterozygous tau (FRP 22 h, triangles) and homozygous tau mutant (FRP 20 h, open circles) *Mesocricetus auratus* maintained under LD 14:10. Mean survival times for wild type, hetero‐ and homozygous strains are 17.5, 10.9, and 15.8 months respectively 90.

Although these studies report a reduction in fitness under non‐24 hours housing, tests of circadian resonance must also examine whether it is the match between endogenous period of the clock and environment that confers advantage, rather than adherence to the “normal” 24 hours cycle. This has been tested in a number of organisms in which mutation in components of the clockwork has yielded phenotypic variation in free running period (FRP; i.e. internal clocks which run with different speeds) (Fig. 3). Ouyang et al. employed this approach by performing competition trials using non‐conjugative strains of *Synechococcus* cyanobacteria maintained under different environmental cycles 80. Crucially, strains with a FRP similar in frequency to the environmental LD cycle were always favored. Furthermore, an arrhythmic strain was more competitive in constant light, yet was consistently undermined by a resonant opponent when maintained in a rhythmic environment (Fig. 3A–C) 81. Similar results were observed in studies involving *Arabidopsis thaliana* 82. Plants with an FRP matching the LD cycle had greater concentrations of chlorophyll, higher carbon fixation, increased aerial biomass, and a larger visible leaf area. In competition trials where plants were grown in close proximity to one another on different lighting schedules the resonant plants out‐competed those in circadian dissonance (Fig. 3D). Hence, the circadian clock in these organisms confers a clear selective advantage when placed in an oscillating environment. However, the fact that arrhythmic strains were favored in constant conditions again suggests that biological rhythmicity is not inherently beneficial.

## The cost of circadian desynchrony in mammals: Insights and open questions

The ability of animals to adapt their behavior to novel or challenging environments, coupled with an internal circadian system comprising multiple semi‐autonomous clocks results in a much more complex resonance relationship with any given environmental zeitgeber. This has made assessing the intrinsic benefits of circadian rhythmicity in mammals much more difficult. For example, under laboratory conditions, rendering an animal arrhythmic by lesioning the SCN carries no deleterious effect in terms of rodent survival or overt pathology 83, 84. However, a series of studies by Decoursey and colleagues found a distinct adverse effect of overt circadian disruption under more natural conditions 85, 86, 87. SCN‐lesioning of two semi‐fossorial rodent species correlated with higher rates of predation and reduced survival, due to the group's greater tendency to leave the burrow at inappropriate times. Hence, selective advantage is driven by the organism's ability to relate to its environment rather than any inherent benefit.

**Figure 4:**
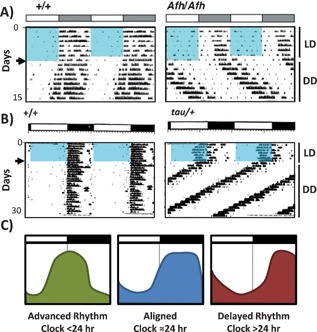
Entrainment to non‐resonant LD cycles leads to an altered phase relationship between behavioral and environmental cycles. **A–B:** Double plotted actograms of wheel‐running activity under LD (12:12 hours, as denoted by black and white bars, blue boxes indicate light phase in (**A**) and DD (arrow indicates release into DD). Short vertical bars represent bouts of wheel running activity. Circadian clocks have an inherent cycle length of ∼24 hours, to match with the day/night cycle of the Earth. Variations in period length of the clock in different organisms (or tissues) can be important in dictating subtle differences in the phase relationship between the exogenous zeitgebers (e.g. solar time) and internal physiological or behavioral rhythms 119. However, as internal and environmental cycles diverge stable yet inappropriate phase alignment can occur – like a watch that runs too fast or too slow; even if it is reset each morning, by the afternoon it is no longer accurate. This is evidenced in wheel‐running activity records of long‐running Afterhours mutant mice (**A**, 116) and short‐running tau mutant hamsters (**B**, 120) housed under 24 hours LD cycles. In both cases, a stable entrainment is achieved, yet the onset of activity is significantly delayed (**A**) or advanced (**B**) relative to the light to dark transition (schematic shown in **C**).

At present, only a limited number of resonance studies have been conducted in laboratory mammals. A reduction in murine longevity when housed in short (4:4 hours LD), but not long (18:18 hours LD) lighting cycles has been reported 88. These observations were not apparent in arrhythmic Per1/2 knockout mice, suggesting the reduced survival was a result of conflict between the external LD cycles and the internal clock. However, the extreme cycle lengths employed, and reliance on gene knockouts, make these studies difficult to interpret. In a particularly interesting set of studies, Ralph and colleagues have taken advantage of the naturally occurring tau mutation in hamsters, to test the impact of non‐resonance on health and longevity. This mutation causes a dose‐dependent acceleration of clock speed (to 20 and 22 hours in homozygotic and heterozygotic animals, respectively) 89. Initial studies showed that survival was reduced in short period mutants maintained in symmetrical 24 hours LD 12:12 conditions 90 (Fig. 3E). However, the effect was only evident in the heterozygous cohort, which could entrain to the 24 hours environment, albeit with an altered phase relationship to the light cycle (see Fig. 4). No reduction in survival was evident in the homozygous tau animals (who simply maintain a free‐running 20 hours period despite the 24 hours LD cycle). Follow‐on studies showed that heterozygous mutant hamsters also develop renal dysfunction and cardiomyopathy with fibrosis and impaired contractility, when housed under 24 hours lighting schedules 91. Importantly however, homozygous individuals, SCN‐lesioned heterozygotes (therefore rendered arrhythmic), and heterozygotes maintained on an LD cycle reflective of their 22 hours FRP showed no such disease profile. These studies imply that maladaptation of the circadian rhythm to a non‐resonant environment is more harmful to the organism, than to have either no behavioral rhythm at all, or one in which the clock free‐runs at its inherent pace despite the fact that it ceases to be predictive of the outside world 92.

Due to the complexity of the mammalian system and the practical limitations of conducting long‐term resonance experiments in laboratory rodents, the exact nature of the circadian dysfunction in these animals is not yet clear. The inappropriate phase relationship established between the behavioral rhythms of the animal and its environment (Fig. 4), instability of the SCN rhythm due to continual resetting by the non‐resonant light cycle, and/or desynchrony between local tissue clocks, and signals coming from the light‐entrained SCN may all contribute to driving a state of internal desynchrony, whereby different clocks of the body are no longer aligned with each other. Similar circadian misalignment may also be a concern for human health, as late chronotype (i.e. a propensity of individuals to wake late and go to bed late) has been associated with elevated BMI, and poor glycemic control in patients with type‐2 diabetes 93, 94, 95.

A number of groups have examined the impact of forced desynchrony on human physiology. Such experiments involve placing subjects under an imposed 20 or 28 hour‐day routine, which includes scheduled sleep bouts and mealtimes, under a dim‐LD cycle. Since the internal clockwork cannot adhere to the extreme cycle length, it free‐runs with its endogenous near 24 hours period. This creates a desynchronized state in which behavioral cycles (eating, sleeping) become disconnected from the clockwork. Sleep disturbance, as well as decreased vigilance and cognitive performance are often observed in desynchronized subjects 96, 97. However, the impact of the forced desynchrony protocol on metabolic parameters and cardiovascular function appear to be particularly profound. Over a relatively short time‐frame (e.g. 10 days) desynchrony protocols have been associated with elevated blood glucose, reduced insulin sensitivity, altered post‐prandial insulin release, reduced circulating leptin, blunted cortisol rhythms, and the advent of hypertension 98, 99, 100. By tracking individuals across the experiment, it is possible to compare physiological responses on days in which the internal clock is aligned with behavioral routine, and those in which the cycles are misaligned. Post‐prandial level of both blood glucose and insulin are elevated to a greater degree during periods of misalignment, indicative of decreased insulin sensitivity 101. Furthermore, by assessing the blood transcriptome in desynchronized subjects, Archer and colleagues recently showed that rhythmic gene expression is strongly attenuated (going from 6.4% of the transcriptome at baseline to only 1.0%) during misalignment 102. Together these studies demonstrate the rapid and profound impact that results from detaching behavioral routine from our internal clockwork.

## Targeting the clockwork: Novel pharmacological tools

**Figure 5:**
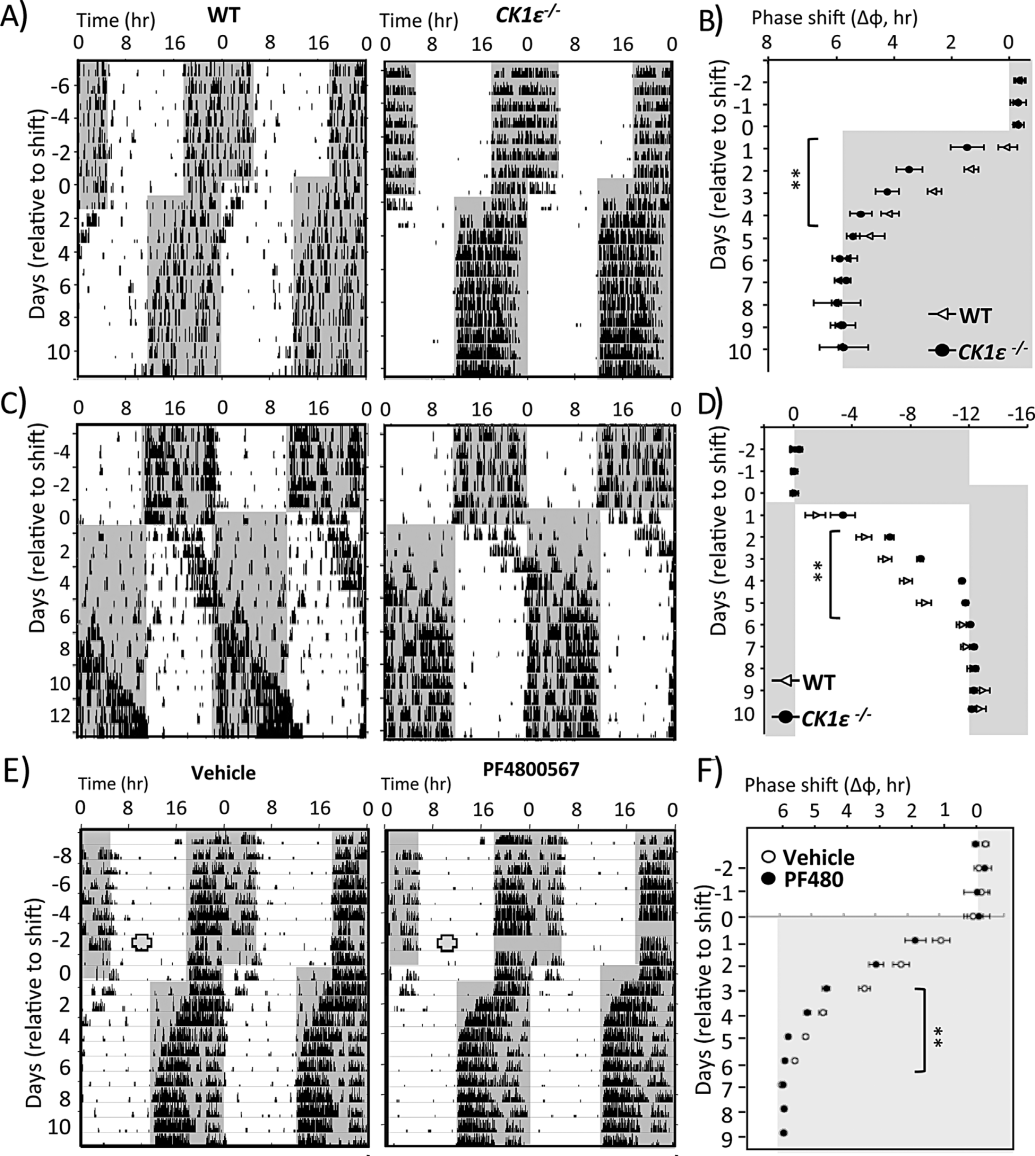
Genetic or pharmacological targeting of CK1e accelerates re‐entrainment to shifts in LD cycle. Rapid Entrainment of Behavioral and Physiological Rhythms to Phase Shifts in CK1ϵ−/− Mice. **A–D**: WT and CK1ϵ−/− mice were subjected to a 6 hours advance (**A** and **B**) or 12 hours delay (**C** and **D**) of the LD cycle. As shown by representative actogram records of wheel‐running locomotor activity (**A** and **C**) or group analysis of activity onset (**B** and **D**), CK1ϵ−/− mice entrained to the new LD phase significantly faster than WT mice. Shading indicates lights‐off. **p < 0.01, repeated‐measures two‐way ANOVA. **E–F:** WT mice were implanted with an osmotic minipump containing either vehicle or the selective CK1e inhibitor, PF4800467. Two days post‐implantation, the mice were subjected to a 6hr phase advance of the LD cycle (**E**), and daily onset of activity was determined each day (**F**). Mice treated with PF4800567 exhibited a significant acceleration in the time required to re‐entrain locomotor activity rhythms to the new LD cycle. Shading indicates lights‐off; red circles indicate the timing of pump implantation. **p < 0.01, repeated‐measures two‐way ANOVA. Adapted from 111.

In parallel with our increased understanding of the clock and its role in pathology, research focus on the clock as a therapeutic target has grown considerably. Circadian‐based interventions such as bright‐light therapy and melatonin administration have long been used to strengthen or modulate circadian rhythms, for example in elderly patients or those with circadian based sleep disorders 103. However, these strategies are now being applied to a wide‐range of conditions, including neurological disorders, neurodegenerative disease, and type‐2 diabetes 104, 105, 106. Moreover, development of pharmacological tools capable of directly modulating the molecular clockwork has greatly expanded. Chemical modulators of casein kinase 1ϵ/δ (CK1ϵ/δ), REVERB, and CRY have all been developed, and have already shown potential benefit in animal models of metabolic disease 107, arrhythmia 108, inflammation 109, and mania 110. Furthermore, we recently demonstrated that targeting of the clock through inhibition of CK1ϵ was effective in enhancing adaption of mice to shifts in the LD cycle, in terms of both behavioral and molecular clock rhythms 111 (Fig. 5). Accelerated re‐entrainment has also been achieved by targeting SCN function by modulating neuropeptide signalling 112, 113. While these studies highlight the progress made towards targeting the clock for therapeutic benefit, realizing this goal will require extreme caution. The pervasiveness of the clock and circadian rhythms across our physiology may make it near impossible to target single pathways without a cascade of knock‐on effects.

Importantly, damping and disruption of behavioral and physiological circadian rhythms are also associated with advanced aging, as well as with a number of pathological conditions. For example, altered circadian rhythms have been identified as an early indication in Alzheimer's disease 114. Similarly, disrupted rhythms are commonly observed in neurological conditions, such as schizophrenia and bipolar depression, and often herald periods of worsening clinical symptoms. Therefore, interventions designed around strengthening or entraining our biological rhythms as a whole, hold great promise, both in clinical conditions associated with circadian disruption and to lessen the impact of our modern desynchronizing society.

## Conclusions

It remains difficult to demonstrate inherent benefits of circadian rhythmicity in mammals. However, a clear detrimental impact occurs when the internal clock is not aligned with either environmental or behavioral rhythms in controlled human and animal studies, suggesting that loss of internal coordination and rhythmicity does have a negative impact on our well‐being. This supports clinical observation studies that linked circadian disruption to pathological states such as obesity, diabetes, immune dysfunction, and cancer 8, 9, 10. Our ability to understand the pathological causes and consequences of circadian disruption in animals and humans should greatly improve over the coming years.

The development of transgenic animals in which we can modulate clock speed in a tissue‐specific manner will allow direct assessment and molecular dissection of the impact of internal desynchrony on physiological homeostasis. Recent development of an in vivo bioluminescence recording cage by Saini et al. 115 allow real‐time measurements of locomotor and gene reporter activity in free‐moving mice over extended periods. Combined with viral delivery of clock‐gene reporters, this technology makes it possible to track the activity of the SCN (assessed through activity profiles) in conjunction with any other virally targeted tissue, and thus allow for the first time perhaps, molecular measurements of internal desynchrony. In the clinical setting, the increased sophistication and availability of remote monitoring tools for recording of behavioral and circadian rhythms in patient cohorts will not only improve data gathering, but also potentially increase our ability to identify markers of desynchrony and other forms of circadian disruption.
